# Lectotypification of ten names of *Carex* hybrids (Cyperaceae), with notes on their morphology, habitat, and distribution

**DOI:** 10.3897/phytokeys.272.189738

**Published:** 2026-03-24

**Authors:** Helena Więcław, Jacob Koopman

**Affiliations:** 1 Institute of Marine and Environmental Sciences, University of Szczecin, Adama Mickiewicza 18, 70-383, Szczecin, Poland Institute of Marine and Environmental Sciences, University of Szczecin Szczecin Poland; 2 ul. Kochanowskiego 27, 73-200 Choszczno, Poland Unaffiliated Choszczno Poland

**Keywords:** *Carex × beckmanniana*, *Carex × csomadensis*, *Carex × fussii*, *Carex × gerhardtii*, *Carex × limnogena*, *Carex × prahliana*, *Carex × schuetzeana*, *Carex × silesiaca*, *Carex × sooi*, *Carex × wolteri*

## Abstract

Lectotypes are designated for ten names of *Carex* hybrids based on material deposited in the B, BP, and WRSL herbaria. Morphological comparison between the hybrids and their parental species is provided, as well as information on their habitat and distribution.

## Introduction

In the genus *Carex* L. (Cyperaceae), which counts over 2000 species (POWO 2026), hybridisation is a relatively common phenomenon (e.g. Cayouette and Catling 1992; Więcław and Koopman 2013; Koopman et al. 2019, 2021, 2023, 2025; Cayouette and Léveillé-Bourret 2021; Řepka 2024). A lot of *Carex* hybrids are sterile (Cayouette and Catling 1992), but others are partially fertile, especially in the sections *Phacocystis* Dumort. (Faulkner 1972) and *Ceratocystis* Dumort. (Schmid 1982). Fertility is one of the factors stabilising hybrid-derived individuals and in effect can give rise to new species, i.e. an independent, morphologically and genetically recognizable, and self-reproducing entity (Mallet 2007; Soltis 2013). Given the importance of hybridisation in plant evolution, accurate identification of hybrid individuals is crucial, e.g. identifying *Carex* hybrids is conclusive to a better understanding of the mechanisms leading to diversification in this species-rich genus (Maguilla and Escudero 2016; Pedersen et al. 2016).

*Carex* hybrids are generally morphologically and genetically intermediate in relation to their parental taxa or they show a mosaic of parental, intermediate and unique characters (e.g, Jermy et al. 2007; Więcław and Wilhelm 2014; Cayouette and Léveillé-Bourret 2021; Nagasawa et al. 2021). Sometimes, *Carex* hybrids with different levels of fertility grow abundantly alongside the parental species and create backcrosses, making their identification even more difficult (e.g. Jermy et al. 2007; Więcław et al. 2023). These issues can lead to taxonomic confusion and misidentification. Therefore, the typification of hybrid names serves as a reference point, showing the morphological boundaries between the parental species and the hybrids, thereby resolving nomenclatural and taxonomic disagreements.

During our study on *Carex* hybrids occurring in Poland (Więcław et al., unpublished data), we encountered in herbaria some original material of so far not typified names. Some of them were described by Figert (1886, 1888, 1889, 1899), while others were collected admittedly by Figert, but the binomial name and description were given by Appel (in Callier 1892) and Junge (1906). In addition, we typified some names of *Carex* hybrids described by Simonkai (1887), Gross (1905), and Jakucs (1953).

## Material and methods

Taxonomic literature, including protologues, as well as specimens deposited at the herbaria of B, BP, GLM, and WSRL, were examined (acronyms follow Thiers 2026, continuously updated). We also consulted two online databases (JACQ Virtual Herbaria 2026; GBIF 2026) to verify the presence of type specimens. The lectotypes were designated by comparing specimens with protologues, with paying special attention to collection date, locality, and collector(s), and selecting the most complete specimens, in accordance with Art. 9.3 of the “Madrid Code” (Turland et al. 2025). The nomenclature follows Koopman (2022).

## Results and discussion

### Carex × beckmanniana

Taxon classificationPlantaePoalesCyperaceae

Figert, Deutsche Bot. Monatsschr. 7: 185 (1889) [ C. riparia × C. rostrata]

C6F23BD0-58E3-54FE-BCFF-B35458B4958A

= Carex × superriparia Hübl, Verh. Zool.-Bot. Ges. Wien 82: 22, 56 (1932).

#### Lectotype designated here.

Poland. Flora von Schlesien. Lüben: Krummlinde, l. cl., 4.6.1889. Leg. E. Figert (B barcode B 10 1334754; isolectotype: GLM barcode GLM-P-0109033) (for image see JACQ Virtual Herbaria; available on http://herbarium.bgbm.org/object/B101334754).

#### Morphology.

This hybrid is more or less intermediate between its parental species, especially in leaf width and distribution of stomata, as well as in length of ligule and utricle (Jermy et al. 2007). *Carex × beckmanniana* has leaves 7–10 mm wide, blue-green, with long, needle-shaped tip. The ligule is 2–5 mm long with a rounded apex, like in *C. riparia*. The stomata in this hybrid are located on both sides of the leaves (amphistomous). The inflorescence is up to 20 cm long with 1–3 male and 2–4 female spikes. The lowest bract is as long as or slightly longer than the inflorescence. The utricles are yellow-green, glabrous, veined, flat, and empty (i.e. with undeveloped nuts), 5.5–6.5 mm long, and gradually tapering into a *ca* 1.5 mm long bifid beak.

#### Habitat.

Swamps, fens, edges of lakes, along slow-flowing rivers and canals.

#### Distribution.

Widespread in Europe and known from Belgium, Denmark, Estonia, Finland, France, Germany, Great Britain, Italy, Latvia, Netherlands, Norway, Poland, Sweden, and Switzerland (Koopman 2022).

### Carex × csomadensis

Taxon classificationPlantaePoalesCyperaceae

Simonk., Enum. Fl. Transsilv.: 556 (1887) [C. riparia × C. vesicaria]

4D13D3B3-A2C0-5139-A8D9-12247FC8E213

#### Lectotype designated here.

**Figure 1.**
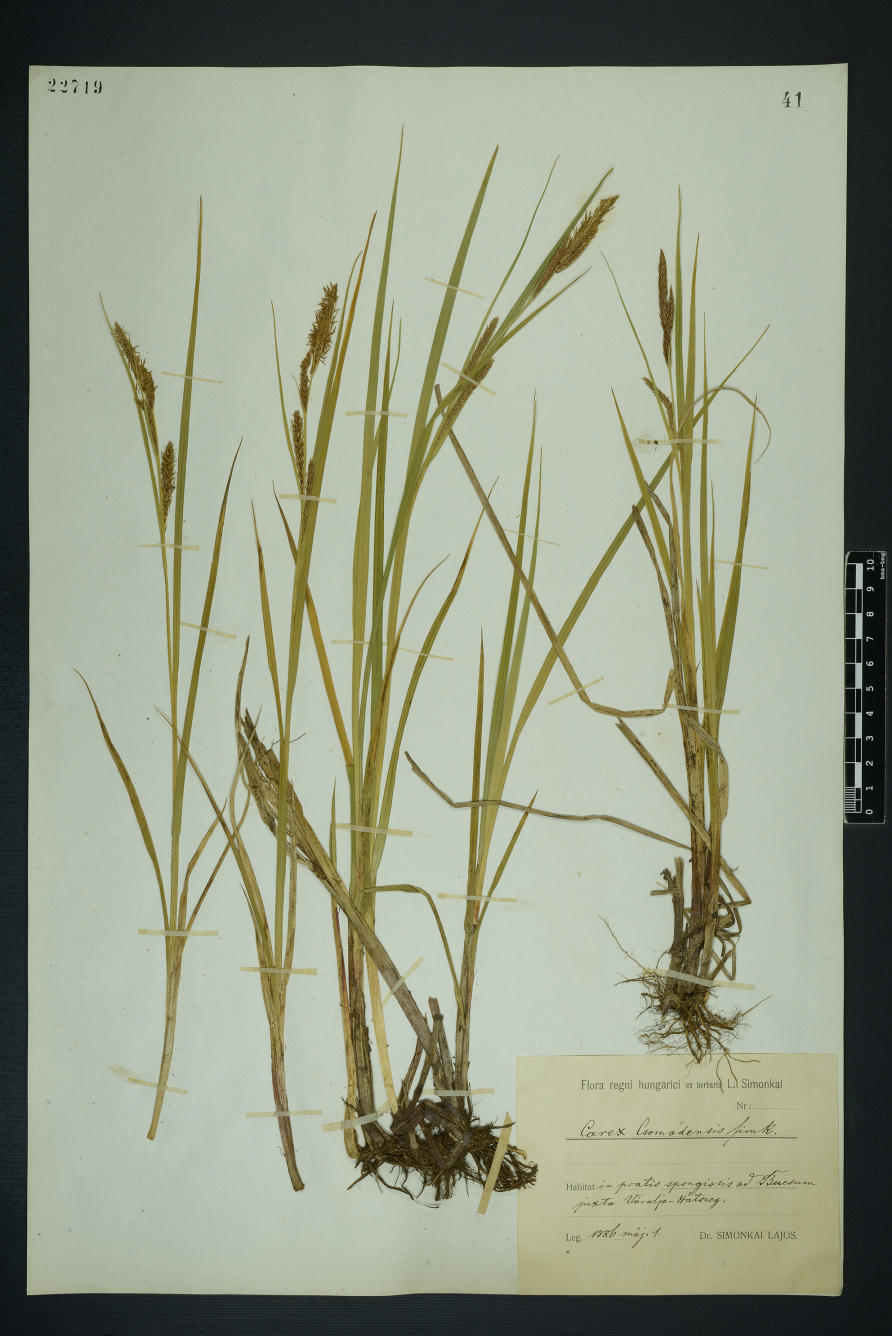
The lectotype of *Carex × csomadensis* Simonk. (BP barcode 22719). Image used with permission from the Herbarium BP, Hungarian Natural History Museum, Budapest.

Hungary. Flora regni hungarici ex herbario L. Simonkai, Habitat in pratis spongiosis ad Buesum iuxta Váralja-Hátszeg. 1886 maj. 1. Leg. L. Simonkai (BP barcode 22719) (Fig. 1).

#### Morphology.

Usually, this hybrid is morphologically closer to *C. riparia* (Jermy et al. 2007). However, *C. × csomadensis* has narrower leaves (4–6 mm broad), shorter male spikes (*ca* 3 cm) and more strongly veined and shiny utricles than *C. riparia*. From *C. vesicaria* it can be distinguished by its leaf colour (glaucous rather than yellow-green), its broader male spikes (3–4 mm), and the darker, shorter utricles. The inflorescence is 15–30 cm long with 2 male spikes and 2–3 female spikes; the lowest female spike is usually pedunculate. The lowest bract is as long as or slightly longer than the inflorescence. The utricles are 5–6 mm long, brown, ± shiny, strongly veined, and sterile. The beak is widely bifid.

#### Habitat.

Swamps, fens, and edges of lakes, along slow-flowing rivers and canals.

#### Distribution.

Widespread in Europe and known from Austria, Czech Republic, Denmark, Estonia, France, Germany, Great Britain, Hungary, Ireland, Italy, Netherlands, Norway, Northwest European Russia, Poland, Romania, Sweden, and Ukraine (Koopman 2022).

### Carex × fussii

Taxon classificationPlantaePoalesCyperaceae

Simonk., Enum. Fl. Transsilv.: 548 (1887) [C. elongata × C. paniculata]

ACE6B257-2634-5998-A944-81AEB66CD6D5

≡ Vignea × fussii (Simonk.) Soják, Cas. Nár. Mus., Odd. Prír. 148: 195 (1979 publ. 1980).
= Carex × belezii H.Lév. & Vaniot, Bull. Assoc. Franç. Bot. 5: 145 (1903).

#### Lectotype designated here.

**Figure 2.**
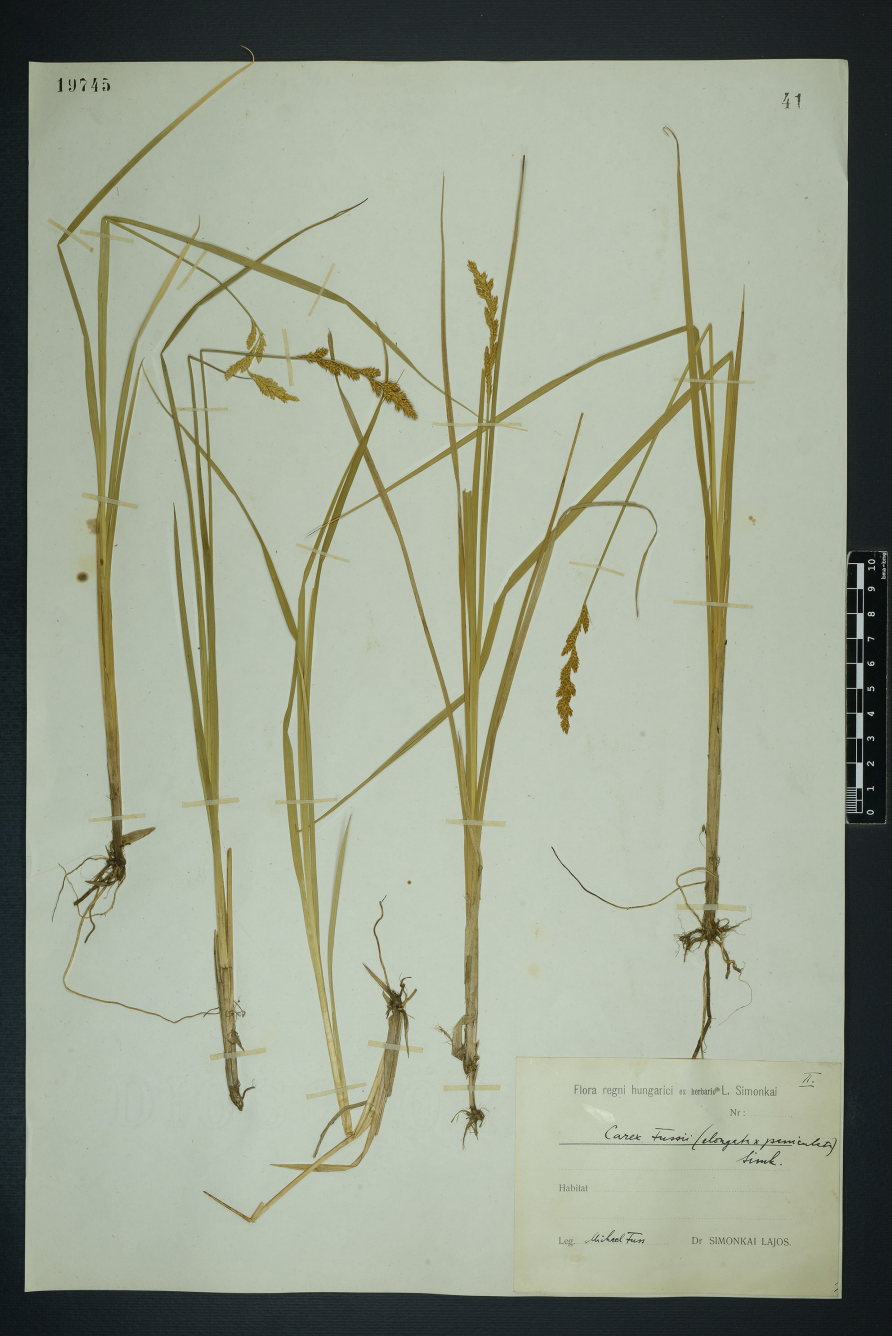
The lectotype of *Carex × fussii* Simonk. (BP barcode 19745). Image used with permission from the Herbarium BP, Hungarian Natural History Museum, Budapest.

Hungary. Flora regni hungarici ex herbario L. Simonkai, Leg. M. Fuss (BP barcode 19745) (Fig. 2).

#### Note.

There is no location or date given on the herbarium sheet, but Simonkai (1887) mentioned that he received the hybrid from Mihály Fuss in 1882, who collected it near the village of Vestény in Hungary.

#### Morphology.

Intermediate between the parental species. From *C. elongata* it can be distinguished by its more massive and stiff culms, its wider, brown to red-brown scale-like basal sheaths, and its usually shorter and less veined utricles. It differs from *C. paniculata* by its shorter inflorescence and red-brown female glumes, shorter than the utricles. The inflorescence is 4–7(–8) cm long with bisexual spikes (female at the top and male at the base). The utricles are 3–4 mm long, brown, and veined. The beak is *ca* 1 mm long, truncate or notched.

#### Habitat.

Swamp forests and along lake shores.

#### Distribution.

This hybrid is known from France, Germany, Italy, Netherlands, Poland, Romania, Central European Russia, and Ukraine (Koopman 2022).

### Carex × gerhardtii

Taxon classificationPlantaePoalesCyperaceae

Figert, Deutsche Bot. Monatsschr. 4: 153 (1886) [C. echinata × C. remota]

CEF54AFC-9800-5E49-A2FA-A64CAF8FC703

≡ Vignea × gerhardtii (Figert) Soják in Čas. Nár. Mus., Odd. Přír. 148: 195 (1979, publ. 1980).
= Carex × vierhapperi Beck, in Ber. Deutsch. Bot. Ges. 4: CCIX (1887).

#### Lectotype designated here.

**Figure 3.**
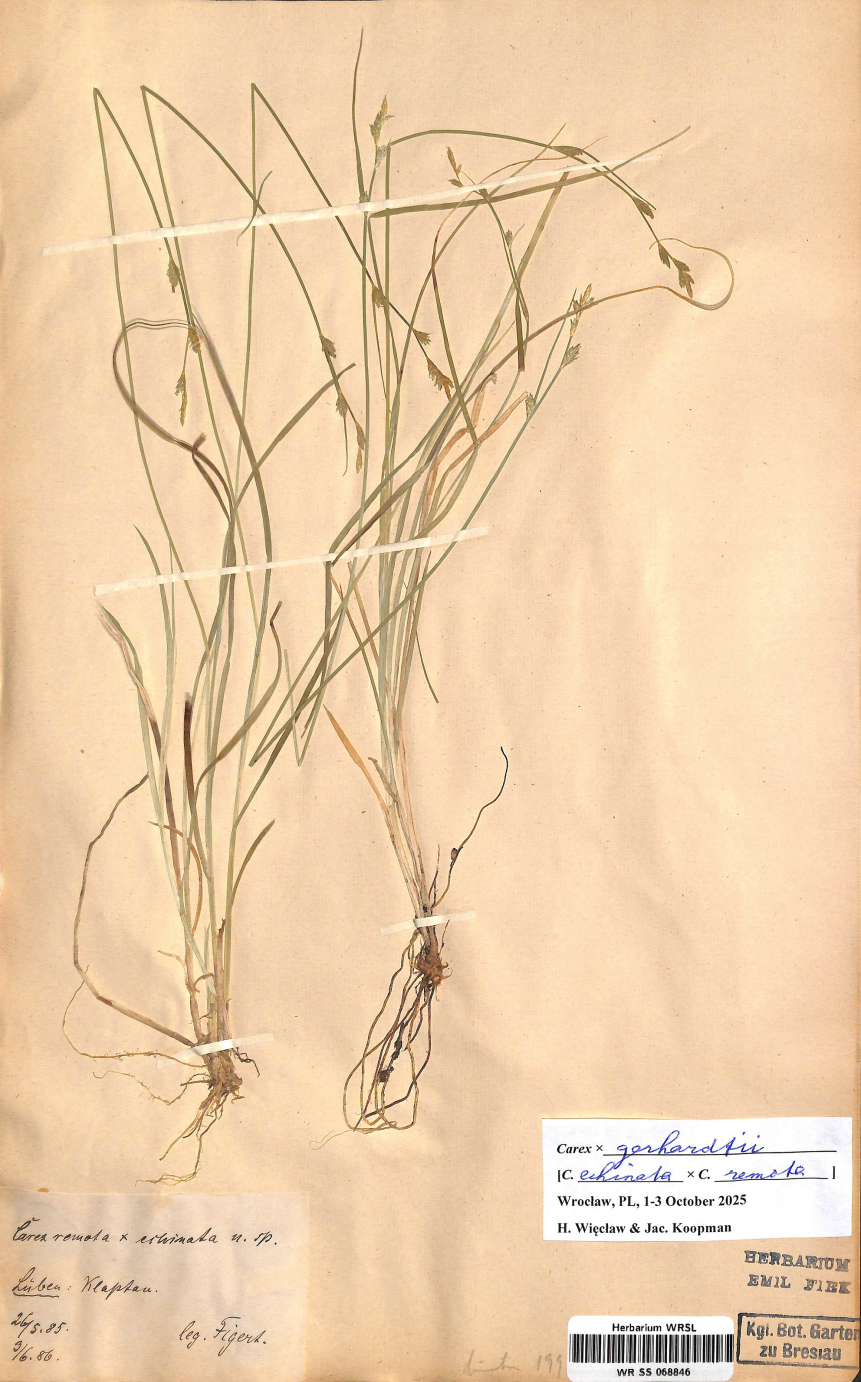
The lectotype of *Carex × gerhardtii* Figert (WRSL barcode WR SS 068846). Image used with permission from the Herbarium WRSL, Museum of Natural History, University of Wrocław, Poland.

Poland. Schlesien. Lüben: Klaptau. 26/5/1885. 3/6/1886. Leg. E. Figert (WRSL barcode WR SS 068846; isolectotype: WA barcode WA0000011112) (Fig. 3).

#### Note.

Figert gave on the label two dates, 26 May 1885 and 3 June 1886. In the protologue is given “von mir entdeckt am 26. Mai 1885” [discovered by me on 26 May 1885]. The protologue was written in October 1886, so, obviously Figert visited the site again on 3 June 1886.

#### Morphology.

This hybrid exhibits a mosaic of parental and intermediate characters. It grows caespitose, like both parental species. The inflorescence is 3–5 cm long, with 5–7 obovoid, bisexual spikes (female at the top, male below); the lowest two spikes are somewhat distant and either entirely female or with a few male flowers at the base. The long lowest bract is inherited from *C. remota*, the toothed beak of the utricle from *C. echinata*. The utricles are empty, *ca* 3 mm long, green, glabrous, oblong-ovoid, with a bifid, serrated beak.

#### Habitat.

Wetlands, usually in swamp forests.

#### Distribution.

It is known from Austria, Germany, Italy, Poland, and Central European Russia (Koopman 2022).

### Carex × limnogena

Taxon classificationPlantaePoalesCyperaceae

Appel, Deutsche Bot. Monatsschr. 10: 168 (1892) [C. appropinquata × C. diandra]

9ABD9270-9363-52C7-A94F-01FE497D91FC

≡ Vignea × limnogena (Appel) Soják, Cas. Nár. Mus., Odd. Prír. 148: 196 (1979, publ. 1980).
= Carex × limnogena f. *superdiandra* Junge, Verh. Naturwiss. Vereins Hamburg, ser. 3, 12: 5 (1904).
= Carex × limnogena f. *superparadoxa* Junge, Verh. Naturwiss. Vereins Hamburg, ser. 3, 12: 5 (1904).

#### Lectotype designated here.

Poland. A. Callier, Flora silesiaca exsiccata Nr 111. Liegnitz: am “kleinen Grundsee” bei Arnsdorf. 19.6.1889. Leg. E. Figert (B barcode B 10 1334926; isolectotypes: C barcode C.505254; WA barcode WA0000011030 barcode WA0000011031) (for image see JACQ Virtual Herbaria; available on http://herbarium.bgbm.org/object/B101334926).

#### Note.

Appel (in Callier 1892) referred in the protologue to a herbarium sheet with material collected by Figert, but he erroneously gave a reference to “Resultate der Durchforschung der schlesischen Phanerogamenflora im Jahre 1888”, where this hybrid is not listed (see Fiek and Pax 1888). The first mention of this hybrid, as *C. paradoxa × teretiuscula* Figert, collected in Arnsdorf (now Miłkowice in Poland) near Liegnitz (Legnica) is in Fiek (1889).

#### Morphology.

This hybrid is morphologically intermediate between the parents. It is caespitose, less dense than in *C. appropinquata*, but denser than in *C. diandra*. The leaves are very narrow (*ca* 2 mm wide) and shorter than the flowering stems. The inflorescence is about 4 cm long with bisexual spikes (male at the top and female below). The utricles are empty, *ca* 3 mm long, dull green-brown, glabrous, unveined, gradually tapering into a short beak.

#### Habitat.

Wetlands, wet forests in river valleys.

#### Distribution.

This hybrid is known from Austria, Belarus, Czech Republic, Denmark, Finland, France, Germany, Italy, Northwest European Russia, Sweden (Koopman 2022) and Poland (Fiek 1889).

### Carex × prahliana

Taxon classificationPlantaePoalesCyperaceae

Junge, Verh. Naturwiss. Vereins Hamburg, ser. 3, 14: 118 (1906) [C. lasiocarpa × C. rostrata]

5C908C03-AE52-5F3C-BC28-56D1E4EC6429

#### Lectotype designated here.

**Figure 4.**
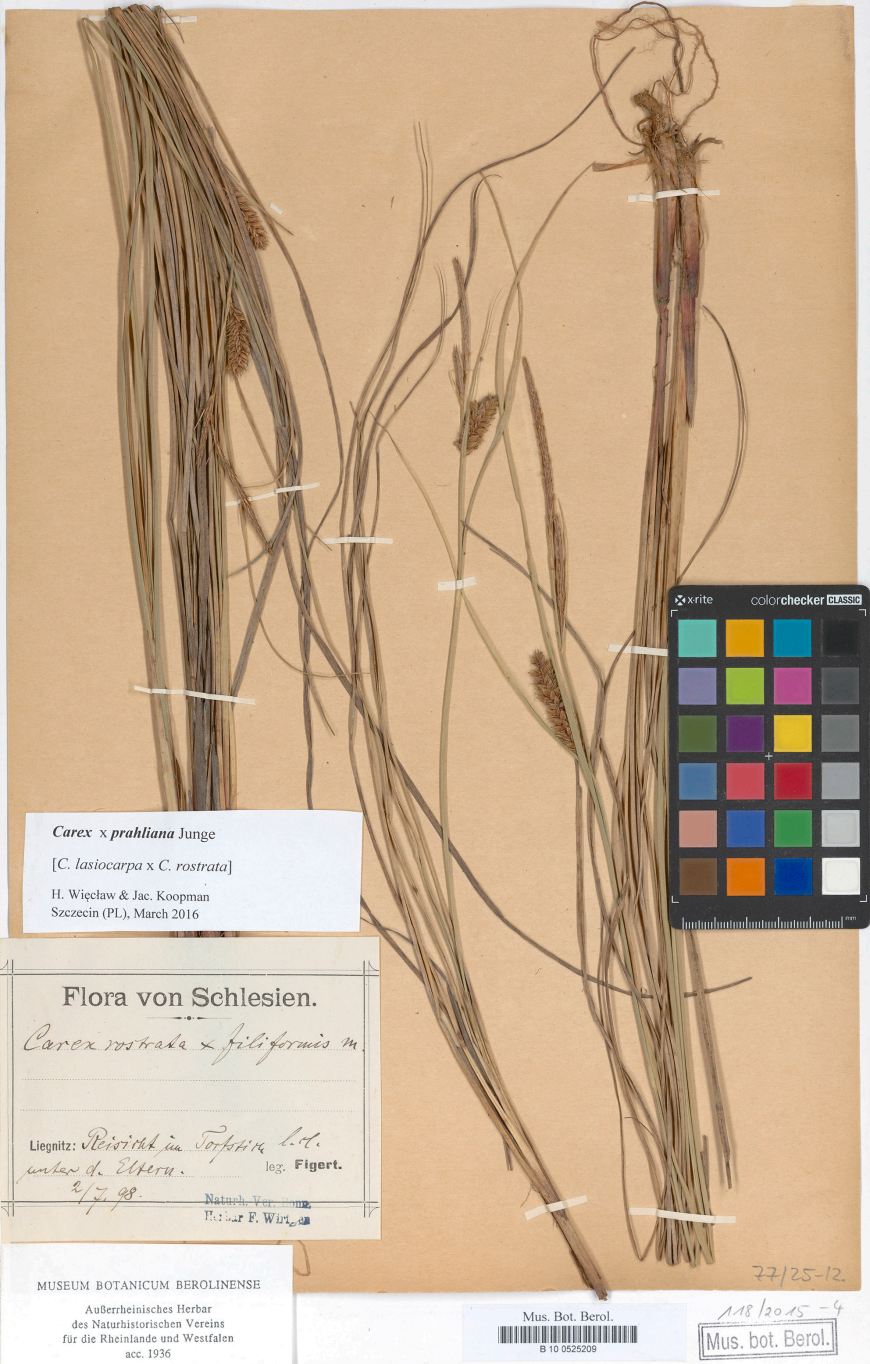
The lectotype of *C. × prahliana* (B barcode B 10 0525209). Image used with permission from the Herbarium B, Botanic Garden and Botanical Museum Berlin, Germany.

Poland. Flora von Schlesien. Liegnitz: Reisicht im Torfstich, unter d. Stammeltern l.cl., 2/7/1898. Leg. E. Figert (B barcode B 10 0525209; isolectotypes: B barcode B 10 0525210, GLM barcode GLM-P-0109021) (Fig. 4).

#### Note.

Junge (1906) referred in the protologue to Figert (1898) who discovered this hybrid as *C. rostrata × filiformis* near Reisicht (now Rokitki in Poland), NW of Liegnitz (Legnica).

#### Morphology.

This rhizomatous hybrid has narrow leaves, usually 2–4 mm wide. The stomata are located on both sides of the leaves (amphistomous). Inflorescence *ca* 14 cm long with 2–3 male spikes and 1–2(–3) female spikes. The lowest bract is longer than the inflorescence. The utricles are green-brown, hairy, veined, 3.5–4.5 mm long, abruptly contracted into a *ca* 1 mm long bifid beak. According to Řepka and Řepka (2025) the utricles of *C. × prahliana* can be partially fertile.

#### Habitat.

Bogs, marshes, swamps, wet forests.

#### Distribution.

This hybrid is known from Finland, France, Germany, Norway, Poland, Northwest European Russia, and Sweden (Koopman 2022). Recently, it has also been found in the Netherlands (Koopman et al. 2025) and in the Czech Republic (Řepka and Řepka 2025).

### Carex × schuetzeana

Taxon classificationPlantaePoalesCyperaceae

Figert, Allg. Bot. Z. Syst. 5: 186 (1899) [C. appropinquata × C. canescens]

BA09A6DF-6145-5834-A2E5-2A3091872BF9

≡ Vignea × schuetzeana (Figert) Dostál, Folia Mus. Rerum Nat. Bohemiae Occid., Bot. 21: 16 (1984).
= Carex × schuetzeana f. *supercanecens* Kük. ex Junge, Verh. Naturwiss. Vereins Hamburg, ser. 3, 12: 8 (1904).
= Carex × schuetzeana f. *superparadoxa* Kük. ex Junge, Verh. Naturwiss. Vereins Hamburg, ser. 3, 12: 8 (1904).

#### Lectotype designated here.

**Figure 5.**
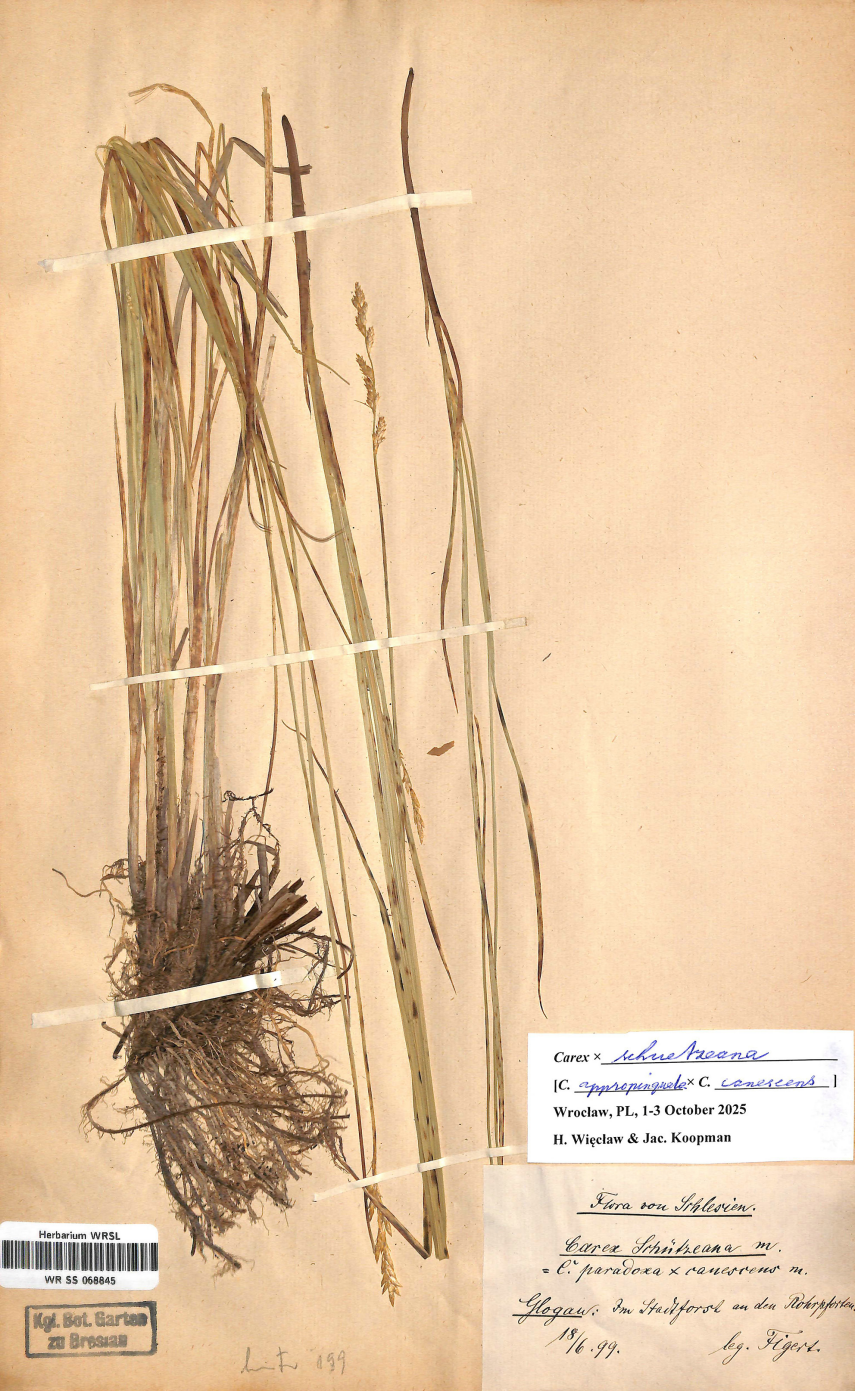
The lectotype of *Carex × schuetzeana* Figert (WRSL barcode WR SS 068845). Image used with permission from the Herbarium WRSL, Museum of Natural History, University of Wrocław, Poland.

Poland. Flora von Schlesien. Glogau, im Stadtforst an den Rohrpforten l. cl., 18/6/1899. Leg. E. Figert (WRSL barcode WR SS 068845; isolectotypes: B barcode B 10 1334762, GLM barcode GLM-P-0109011) (Fig. 5).

#### Morphology.

This hybrid is densely caespitose, like both parents, and in its characters more or less intermediate, but taller than *C. canescens*. The leaves are *ca* 4 mm wide, with stomata on both sides (amphistomous). The inflorescence is short, 3–5 cm long, pale cream-colored, the bisexual spikes with female flowers at the top and male at the base. The empty utricles are dull green-brown, glabrous, unveined, *ca* 2 mm long, and gradually tapering into a beak.

#### Habitat.

Wetlands, wet forests.

#### Distribution.

This hybrid is known from the Czech Republic, France, Germany, Italy, Norway, Poland, and Central European Russia (Koopman 2022).

### Carex × silesiaca

Taxon classificationPlantaePoalesCyperaceae

Figert, Deutsche Bot. Monatsschr. 6: 146 (1888) [C. canescens × C. paniculata]

14EE94F6-3C62-5154-BFA4-B8E0052C07A3

#### Lectotype designated here.

**Figure 6.**
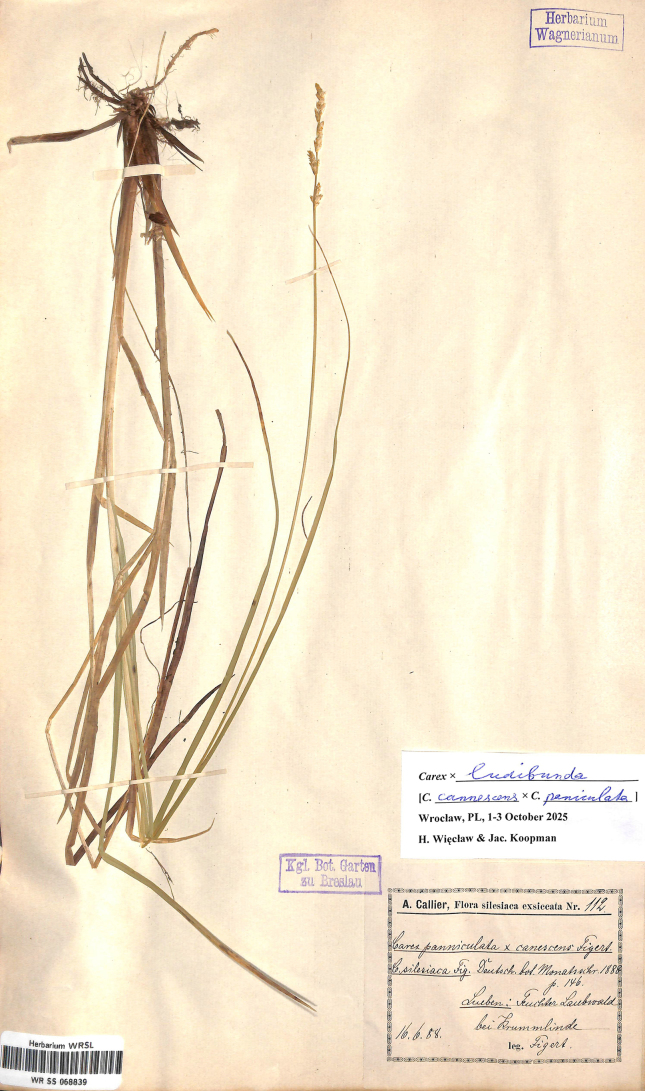
The lectotype of *Carex × silesiaca* Figert (WRSL barcode WR SS 068839). Image used with permission from the Herbarium WRSL, Museum of Natural History, University of Wrocław, Poland.

Poland. A. Callier, Flora silesiaca exiccata Nr 112. Lüben: Feuchter Laubwald bei Krummlinde.16.6.888. Leg. E. Figert (WRSL barcode WR SS 068839) (Fig. 6).

#### Note.

Figert (1888) found this hybrid in the wood near Raszówka (Krummlinde, Kr. Lüben), in Dolnośląskie prov. (Silesia) in Poland and named it *Carex × silesiaca* (as *Carex Silesiaca*). This name, however, is only a synonym of the previously validly described *Carex × ludibunda* J.Gay (Gay 1838).

#### Morphology.

This caespitose hybrid is closer to *C. canescens*, especially in its medium to small stature (Jermy et al. 2007). It differs from *C. canescens* in its more tussocky habit (generally taller), with thicker and broader leaves (2.5–4 mm wide), and rounded ligule. From *C. paniculata* it can be distinguished by its smaller size, pale basal sheaths, short brown-orange inflorescence and its smaller, unwinged utricles. The inflorescence is 4–6 cm long with bisexual spikes (female flowers at the top and male at the base, like in *C. canescens*). The utricles are 2.5–3 mm long, pale green, narrowly ovoid and empty, gradually tapering into a short beak.

#### Habitat.

Marshes and wet swamp forests.

#### Distribution.

This hybrid is rather widespread and known from Denmark, Finland, France, Germany, Great Britain, Italy, Netherlands, Poland, Slovakia, and Central European Russia (Koopman 2022).

### Carex × sooi

Taxon classificationPlantaePoalesCyperaceae

Jakucs, Ann. Hist.-Nat. Mus. Natl. Hung., n.s., 3: 90 (1952 publ. 1953) [C. acutiformis × C. riparia]

9AFC4BC9-2A40-5229-A91C-BE3CEE80C1CB

= Carex × lambertiana H.Lév., Bull. Acad. Int. Géogr. Bot. 21: 268 (1911), nom. illeg.

#### Lectotype designated here.

**Figure 7.**
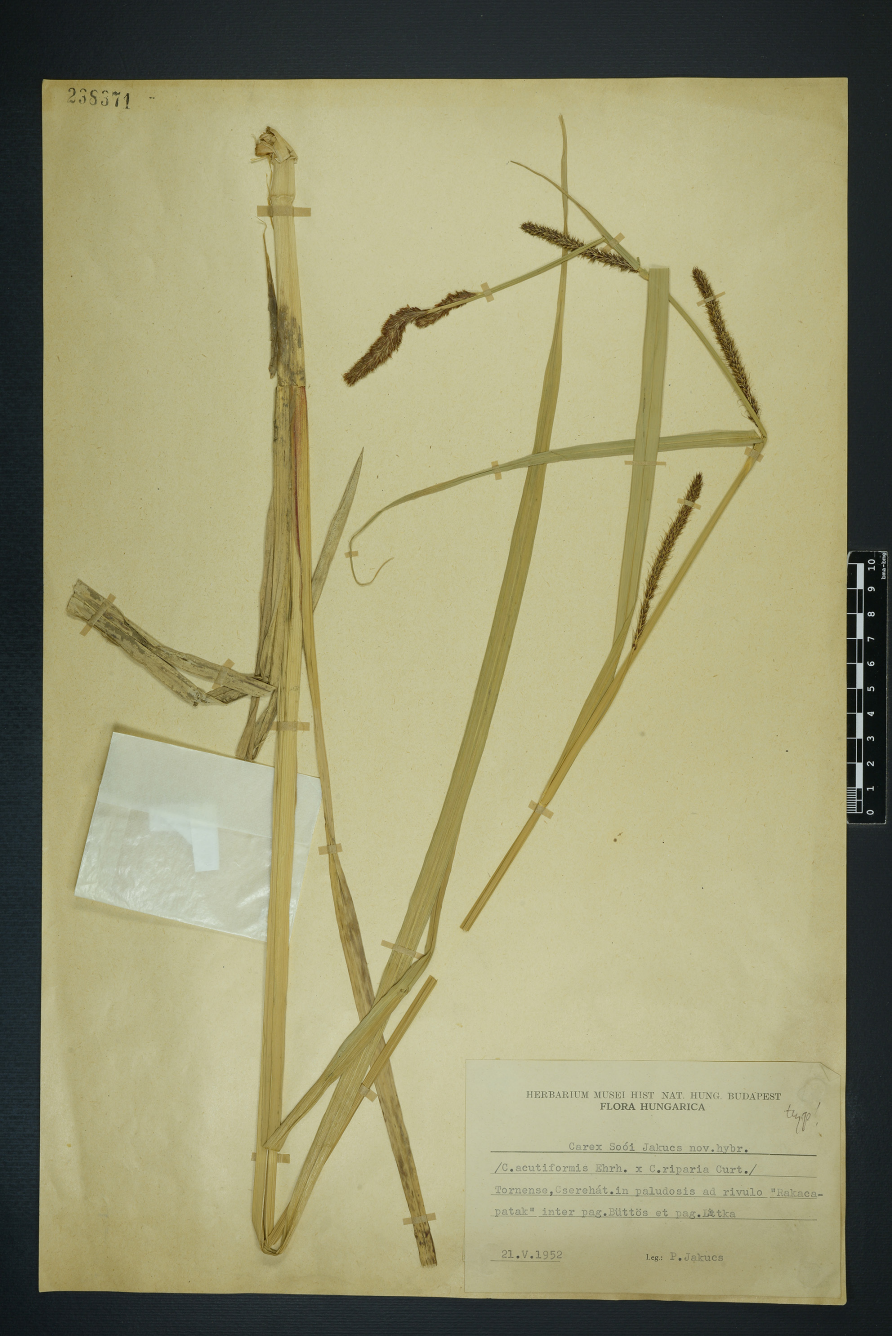
The lectotype of *Carex × sooi* Jakucs (BP barcode 238371). Image used with permission from the Herbarium BP, Hungarian Natural History Museum, Budapest.

Herbarium Musei Hist Nat. Hung. Budapest, Flora Hungarica, Tornense, Cserehát, in paludosis ad rivulo “Rakacapatak” inter pag. Büttös et pag. Littka. 21.V. 1952. Leg. P. Jakucs (BP barcode 238371) (Fig. 7).

#### Morphology.

This hybrid is variable, largely sterile, and occasionally partially fertile (Jermy et al. 2007; Řepka et al. 2024). *Carex × sooi* differs from *C. acutiformis* in its more abruptly tapering leaves, the longer female spikes (5–8 cm) and the acuminate male glumes. From *C. riparia* it differs in its acute ligule, cylindric male spikes, shorter female glumes and shorter utricles and beaks. The inflorescence is (10–)20–30 cm long with 2–3(–4) male spikes and 2–4 female spikes (the lowest sometimes long-pedunculate). The lowest bract is as long as, or slightly shorter than, the inflorescence. The utricles are 4–5.5 mm long, veined, brown, glabrous, gradually tapering into a short, 0.5–0.7 mm long beak.

#### Habitat.

Wetlands, swamps, floodplain forests.

#### Distribution.

This hybrid is known from Belarus, France, Great Britain, Hungary, Latvia, Lithuania, Netherlands, Poland, and Sweden (Koopman 2022). Recently it has also been found in Austria and in the Czech Republic (Řepka et al. 2024).

### Carex × wolteri

Taxon classificationPlantaePoalesCyperaceae

Gross, Allg. Bot. Z. Syst. 11: 23 (1905) [C. pseudocyperus × C. vesicaria]

492B8504-E5E9-5905-B498-60564A942457

#### Lectotype designated here.

Poland. Ex herb. R. Gross. (B barcode B 10 0296538) (for image see JACQ Virtual Herbaria; available on http://herbarium.bgbm.org/object/B100296538).

#### Note.

There is no location or date given on the herbarium sheet, but according to the protologue, Gross (1905) found this hybrid in June 1896 near Tiegenhof (Nowy Dwór Gdański), Poland. In addition, on the label is written mh (mihi), which shows that it was Rudolf Gross who named this hybrid *C. × wolteri* (*Pseudo-cyperus* × vesicaria = *C. wolteri*).

#### Morphology.

This hybrid is more or less intermediate between its parental species. The leaves are 4–5 mm wide and have stomata on the lower surface (hypostomous), like both parents. The inflorescence is 15–20 cm long with 2–3 male spikes and 1 or 2 female spikes. *Carex × wolteri* differs from *C. vesicaria* by its longer female spikes, aristate and ciliate male and female glumes, shorter utricles with a deeply bifid beak. From *C. pseudocyperus* it can be distinguished by its shorter lowest bracts (in the hybrid, the bracts are as long as or slightly longer than the inflorescence) and shorter, usually sessile 1 or 2 female spikes. The empty utricles are usually dull green-brown and glabrous, 4–5 mm long with a 1.5–2 mm long, deeply bifid beak.

#### Habitat.

Wetlands, river valleys, swamp forests.

#### Distribution.

This hybrid is known from Austria, Belarus, Finland, France, Germany, Italy, Norway, Poland, Northwest and Central European Russia, Slovakia, Sweden, and Switzerland (Koopman 2022).

## Supplementary Material

XML Treatment for Carex × beckmanniana

XML Treatment for Carex × csomadensis

XML Treatment for Carex × fussii

XML Treatment for Carex × gerhardtii

XML Treatment for Carex × limnogena

XML Treatment for Carex × prahliana

XML Treatment for Carex × schuetzeana

XML Treatment for Carex × silesiaca

XML Treatment for Carex × sooi

XML Treatment for Carex × wolteri

## Additional information

### Use of AI

No use of AI was reported.

### Author ORCIDs

Helena Więcław https://orcid.org/0000-0002-8165-6532

Jacob Koopman https://orcid.org/0000-0002-8235-1108

### Data availability

All of the data that support the findings of this study are available in the main text.
